# Prognostic Factors for Patients with Small-Cell Lung Cancer Treated with Chemoimmunotherapy: A Retrospective Multicenter Study

**DOI:** 10.3390/curroncol31110482

**Published:** 2024-10-23

**Authors:** Takashi Hatori, Takeshi Numata, Toshihiro Shiozawa, Manato Taguchi, Hirofumi Sakurai, Tomohiro Tamura, Jun Kanazawa, Hiroaki Tachi, Kyoko Kondo, Kunihiko Miyazaki, Norihiro Kikuchi, Koichi Kurishima, Hiroaki Satoh, Nobuyuki Hizawa

**Affiliations:** 1 Department of Respiratory Medicine, Faculty of Medicine, University of Tsukuba, Tsukuba 305-8575, Ibaraki, Japan; s2330424@u.tsukuba.ac.jp (T.H.);; 2 Divisions of Respiratory Medicine, Mito Medical Center, Ibarakimachi 311-3193, Ibaraki, Japan; 3 Division of Respiratory Medicine, Moriya Daiichi General Hospital, Moriya 302-0102, Ibaraki, Japan; 4 Division of Respiratory Medicine, Ibaraki Seinan Medical Center Hospital, Sakai 306-0433, Ibaraki, Japan; 5 Respiratory Center, Ibaraki Prefectural Central Hospital, Kasama 310-8555, Ibaraki, Japan; 6 Department of Respiratory Medicine, National Hospital Organization, Ibaraki Higashi National Hospital, Tokai-Village 319-1113, Ibaraki, Japan; 7 Divisions of Respiratory Medicine, Hitachi General Hospital, Hitachi 317-0077, Ibaraki, Japan; 8 Division of Respiratory Medicine, Ryugasaki Saiseikai Hospital, Ryugasaki 301-0854, Ibaraki, Japan; 9 Division of Respiratory Medicine, Kasumigaura Medical Center, Tsuchiura 300-8585, Ibaraki, Japan; 10 Division of Respiratory Medicine, Tsukuba Medical Center Hospital, Tsukuba 305-8558, Ibaraki, Japan; 11 Division of Respiratory Medicine, Mito Kyodo General Hospital, Mito 310-0015, Ibaraki, Japan

**Keywords:** small cell lung cancer, immunotherapy, prognosis, albumin, liver metastasis, immune-related adverse event

## Abstract

Background: This study aimed to investigate prognostic factors for predicting the survival of patients with extensive-disease-stage small-cell lung cancer treated with chemoimmunotherapy. Methods: Patients were classified according to overall survival (OS): favorable corresponded to an OS ≥ 24 months, moderate corresponded to an OS of 6–24 months, and poor corresponded to an OS < 6 months. Multivariate Cox regression analyses were used to evaluate prognostic factors. Results: Of 130 patients, the proportions of performance status decline and liver metastasis were significantly higher in the poor-prognosis group. With regard to the laboratory findings, neutrophil/lymphocyte ratios and albumin levels differed significantly among the groups. Multivariate analysis showed that the independent prognostic factors for OS were liver metastasis and decreased albumin levels (<3.5 mg/dL). After classifying the patients into three groups according to the quantities of these prognostic factors, the OS differed significantly among the groups (18.3 vs. 13.5 vs. 3.8 months; *p* < 0.001). The incidence of immune-related adverse events (irAEs) was higher in patients without these prognostic factors than in those with both (36% vs. 5%; *p* = 0.01). Conclusion: Liver metastasis and decreased albumin levels are independent unfavorable prognostic factors. Patients with both prognostic factors showed unfavorable OS; however, patients without these factors may have a favorable prognosis but be at greater risk of irAEs.

## 1. Introduction

Small-cell lung cancer (SCLC) is characterized by a more rapid progression than other histological types and is highly associated with smoking [1]. SCLC can be classified into two stages in terms of the choice of chemoradiotherapy or chemotherapy: limited disease (LD) and extensive disease (ED). LD-SCLC is defined as disease that is confined to the thorax, including the ipsilateral hilar, bilateral mediastinal, and bilateral supraclavicular lymph nodes, for which treatment with thoracic irradiation is feasible. In contrast, ED-SCLC is defined as having distant metastasis or extending beyond the area for which thoracic radiation treatment is feasible. Because of the rapid tumor growth, more than two-thirds of patients with SCLC are diagnosed with ED. Combination chemotherapy with platinum and etoposide (ETP) has been positioned as the standard regimen for treating ED-SCLC. Although first-line chemotherapy shows high chemosensitivity, most patients relapse soon after treatment completion; thus, the prognosis of ED-SCLC is poor, with a median overall survival (OS) of less than 12 months [2,3]. Moreover, little progress has been made in treating ED-SCLC for over 20 years.

Recent pivotal trials using immune checkpoint inhibitors (ICIs) have led to a paradigm shift in ED-SCLC treatment. Impower133, a randomized, phase III trial, investigated the efficacy and safety of adding atezolizumab, an anti-programmed death ligand-1 (PD-L1) monoclonal antibody, to a combined carboplatin (CBDCA) and ETP regimen. Both progression-free survival (PFS) and OS were longer in the atezolizumab plus CBDCA and ETP therapy group compared with the control group treated with a placebo plus CBDCA and ETP (OS: hazard ratio [HR] = 0.75; 95% confidence interval [CI], 0.54–0.91; *p* = 0.007; PFS: HR = 0.77; 95% CI, 0.62–0.96, *p* = 0.02) [4]. In the phase III CASPIAN trial, a combination of platinum (cisplatin or CBDCA) and ETP plus durvalumab, an anti-PD-L1 monoclonal antibody, significantly prolonged OS compared to the control group, which was treated with platinum and ETP alone (HR = 0.73; 95% CI, 0.59–0.91; *p* = 0.0047) [5]. Thus, chemoimmunotherapy with combined platinum and ETP plus ICIs is the new standard treatment in the first-line setting of ED-SCLC.

A novel insight from these trials is that some patients treated with chemoimmunotherapy are long-term responders. In the IMpower133 trial, a survival difference of 13% was observed at 18 months between the atezolizumab group and the placebo group (34% in the atezolizumab group vs. 21% in the placebo group) [6]. In the CASPIAN trial, the 3-year OS rate in the durvalumab arm was approximately 17.6%, while it was only 5.8% in the control arm [7]. Although these results suggest that some patients will achieve a durable survival benefit with the addition of ICIs, others will experience relapses, and their prognosis will remain dismal. Therefore, identifying the prognostic factors helpful in predicting the survival of ED-SCLC patients treated with chemoimmunotherapy regimens is required. In non-small-cell lung cancer (NSCLC), tumor mutation burden (TMB) and the expression status of PD-L1 have been reported to be predictive factors of ICI treatment [8,9,10]; however, in contrast to the findings for NSCLC, previous studies showed that neither TMB nor PD-L1 expression status could predict the efficacy of ICI treatment for ED-SCLC [4,11,12].

Based on this background, we conducted a multi-institutional retrospective cohort study including 11 institutions in Ibaraki prefecture, Japan. This study was conducted to investigate prognostic factors helpful in predicting survival for patients with ED-SCLC treated with chemoimmunotherapy regimens.

## 2. Materials and Methods

### 2.1. Ethical Approval

The protocol of this study was approved by the institutional review board of Tsukuba University Hospital (approval number: R04-048). Owing to the retrospective nature of the analysis conducted, the requirement of informed consent from patients was waived. Instead, opt-out statements were published on the websites of each participating institution. This study did not receive funding from any for-profit or not-for-profit organizations or funding agencies.

### 2.2. Patients and Data Collection

Between September 2019 and May 2022, patients who were undergoing a chemoimmunotherapy regimen (Impower133 or CASPIAN regimen) were enrolled in this study. The data cut-off date was 30 September 2022. The inclusion criteria of this study were (1) pathologically diagnosed SCLC and (2) having undergone at least one cycle of a chemoimmunotherapy regimen. To evaluate clinical characteristics affecting survival, we classified enrolled patients into three groups based on previous reports [13,14]. Briefly, patients with an OSs of more than 24 months, 6–24 months, and less than 6 months were classified into favorable-, moderate-, and poor-prognosis groups, respectively.

We collected clinical data at the initiation of the chemoimmunotherapy regimen as baseline. The collected data included the following: age; sex; performance status (PS); smoking status (current, former, or never smoker); clinical stage; the presence of metastases in the brain, liver, or bone; and the history of radiotherapy before chemoimmunotherapy. We also collected the following laboratory data: counts of white blood cells and their fractions and levels of hemoglobin, platelets, albumin, lactate dehydrogenase (LDH), and progastrin-related protein (ProGRP). The cut-off values were defined as the upper limit of the normal value. The absolute number ratio of neutrophils to lymphocytes was calculated as the neutrophil-to-lymphocyte ratio (NLR), and its cut-off was defined as 5.0, as reported previously [15,16].

Each attending physician evaluated antitumor response using CT scans of the chest and abdomen and magnetic resonance imaging of the head. These evaluations were made according to the Response Evaluation Criteria in Solid Tumors version 1.1. Adverse events (AEs) were evaluated according to the Common Terminology Criteria for Adverse Events version 5.1.

### 2.3. Statistical Analysis

Chi-squared or Fisher's exact tests were applied in group comparisons of categorical variables. Continuous variables were compared using Mann–Whitney U tests. The Kaplan–Meier method was used to estimate median PFS and OS, and the log-rank test was applied to compare survivals among groups. The Cox regression model was used to investigate prognostic factors. Variables for multivariate analysis were selected based on their clinical significance and the results of univariate analysis. Hazard ratios in the multivariate analysis are reported with their 95% CIs. Statistical analyses were performed using IBM SPSS statistics (version 24.0) for Windows (IBM Corp., Armonk, NY, USA). All tests were two-sided, with *p*-values < 0.05 considered to indicate statistical significance.

## 3. Results

### 3.1. Patient Characteristics and Efficacy

In total, 130 patients from 11 institutions were enrolled. Table 1 shows the patients’ characteristics at baseline. The median age was 71 years old (range, 42–85). Most patients were current or former smokers. Approximately 25% of the patients had a PS ranging from 2 to 4. The liver was the most frequent metastasis site (40/130, 31%). Regarding platinum doublet regimens, most patients received a CBDCA-based regimen. With respect to baseline characteristics, there were statistically significant differences in liver metastases and a PS decline at 2–4 across the groups, both of which were more frequent in the poor-prognosis group (*p* = 0.03 for liver metastasis, *p* = 0.02 for PS).

The median follow-up period was 9.3 months (95% CI, 6.7–12.9). In the entire population, the objective response rate and disease control rate were 61% (95% CI, 52–69) and 82% (95% CI, 75–89), respectively. The estimated median PFS was 6.4 months (95% CI, 5.8–7.1), and the 1-year PFS rate was 24%. The estimated median OS was 14.4 months (95% CI, 11.2–17.6). The 1-year OS rate was 55%.

### 3.2. Laboratory Data at Baseline

**Figure 1:**
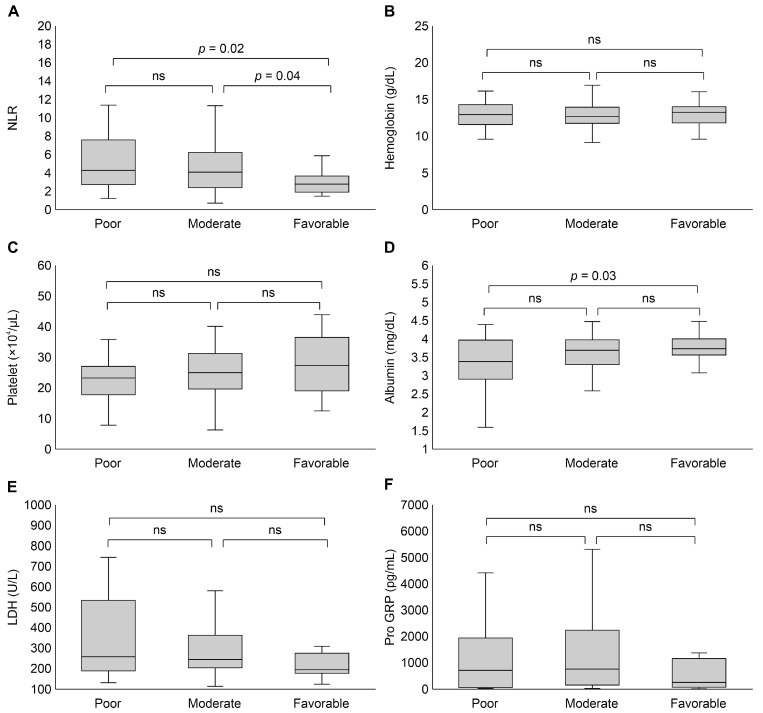
Group comparison of pretreatment laboratory data. (**A**) NLR, (**B**) hemoglobin, (**C**) platelet, (**D**) albumin, (**E**) LDH, and (**F**) ProGRP. Abbreviations: LDH, lactate dehydrogenase; NLR, neutrophil/lymphocyte ratio; ns, not significant; ProGRP, progastrin-releasing peptide.

We compared the laboratory data at baseline among the groups to identify the laboratory parameters affecting OS (Figure 1). The NLR was significantly lower in the favorable-prognosis group compared with that for the other two groups (Figure 1A). A statistically significant difference was also observed in serum albumin levels between the poor- and favorable-prognosis groups (*p* = 0.03, Figure 1D). There was no significant difference in other laboratory data among the groups.

### 3.3. Univariate and Multivariate Analyses

Table 2 presents the results of the univariate and multivariate analyses of OS. The univariate analysis showed that the presence of liver metastasis and albumin levels that have decreased to less than 3.5 mg/dL were associated with poor OS, while an NLR < 5 was significantly associated with favorable OS. Multivariate analysis demonstrated that liver metastases (*p* = 0.002, hazard ratio [HR]: 2.03, 95% CI: 1.25–3.30) and decreased albumin levels (*p* = 0.02, HR: 1.84, 95% CI: 1.14–2.96) were independent unfavorable prognostic factors associated with OS.

### 3.4. Number of Prognostic Factors and the Impact on Survival

**Figure 2:**
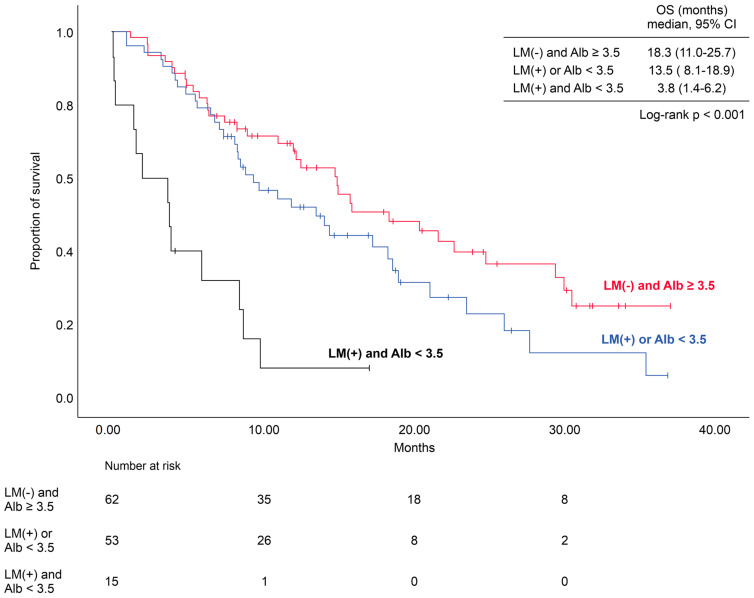
Kaplan–Meier survival curve according to the presence or absence of the prognostic factors. The following is a description of what each line indicates: red, patients without LM and Alb ≥ 3.5 mg/dL; blue, patients with LM or Alb < 3.5 mg/dL; black, patients with both LM and Alb < 3.5 mg/dL. Abbreviations: Alb, albumin; LM, liver metastasis; OS, overall survival.

We then tested the impact of the number of applicable prognostic factors on survival (Figure 2). The OS of patients with both liver metastasis and decreased albumin levels was 3.8 months, which was significantly shorter than that of patients with none or only one of these factors (*p* < 0.001). The OS of patients without these factors tended to be longer than that of patients with one of these factors.

### 3.5. Number of Prognostic Factors and the Impact on Immune-Related Adverse Event (irAE) Incidence

**Figure 3:**
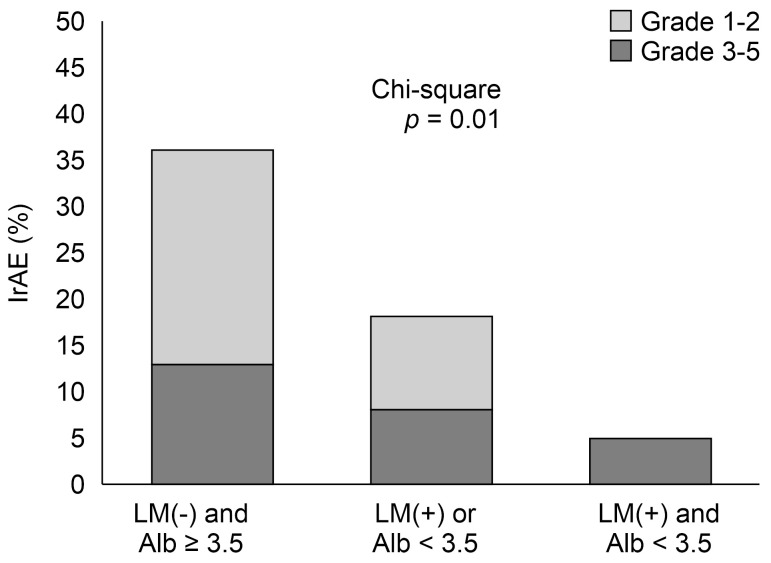
Comparison of the proportion of irAE incidence across the groups. Abbreviations: Alb, albumin; LM, liver metastasis; irAE, immune-related adverse event.

We further evaluated the relationship between the number of applied prognostic factors and the incidence of irAEs (Figure 3). In the overall population, pneumonitis was the most frequent irAE, at 6%, followed by thyroid dysfunction (4%) and hepatitis (3%). The frequencies of all grades of irAEs in patients with neither liver metastasis nor decreased albumin levels, either one, or both were 36%, 18%, and 5%, respectively, with a statistically significant difference among the groups (*p* = 0.01).

### 3.6. Subsequent Chemotherapy

At the cut-off date, 113 patients discontinued their chemoimmunotherapy regimens (97 because of disease progression and 16 because of AEs). Among them, 62 patients received subsequent chemotherapy: 39 patients were administered amrubicin; 19 patients received platinum doublet regimens, including the re-administration of their first-line regimens; and 4 patients received single-agent chemotherapy, such as irinotecan and nogitecan treatment.

## 4. Discussion

In this study, we investigated prognostic factors for predicting the survival of patients with ED-SCLC undergoing chemoimmunotherapy regimens. Our results showed that liver metastasis and decreased albumin levels were independent unfavorable prognostic factors for OS. When the patients were classified based on the number of these prognostic factors, the OS differed significantly among groups. Additionally, the incidence of irAEs was more frequent in patients to which these factors did not apply, suggesting that the evaluation of these prognostic factors could also help predict the risk of irAEs. Because both liver metastasis and serum albumin levels are clinical parameters routinely assessed in diagnostic workups, the results of this study can be easily applied in clinical practice.

This study reveals that liver metastasis is an independent unfavorable prognostic factor of ED-SCLC. In regard to NSCLC, several previous studies reported that patients with liver metastasis had inferior responses to ICI regimens compared with patients with other-organ metastasis [17,18,19,20]. It remains unclear, however, whether liver metastasis affects the survival of patients with ED-SCLC treated with chemoimmunotherapy regimens. A previous study on a large ED-SCLC cohort reported that OS for patients with liver metastases was inferior to that for patients without liver metastasis (9.0 vs. 12.0  months, *p* < 0.001) [21]; however, this study was conducted before ICI-containing regimens were introduced for treating ED-SCLC. The results of this study indicate the necessity of paying attention to the presence of liver metastasis as an unfavorable prognostic factor for chemoimmunotherapy for ED-SCLC as well as NSCLC.

Serum albumin is a nutritional parameter commonly associated with cancer cachexia. Several previous studies reported prognostic significances of pretreatment albumin levels as prognostic factors of both NSCLC and SCLC [22,23,24]; however, most of these studies investigated the significance of albumin levels combined with other laboratory parameters such as albumin-to-alkaline phosphatase ratios, albumin-to-fibrinogen ratios, and albumin/globulin ratios. In this cohort study, we evaluated the significance of albumin alone, considering the simplicity of recording this parameter in daily clinical practice, and identified it as an unfavorable prognostic factor. We thus propose that albumin is a useful laboratory parameter for predicting survival in ED-SCLC.

In our study, we did not identify an independent favorable prognostic factor for patients with ED-SCLC treated with chemoimmunotherapy regimens. NLRs did not reveal statistically significant differences in multivariate analysis, although the NLR was lower in the favorable-prognosis group compared with that of moderate- or poor-prognosis groups. One way to interpret the results is based on the cut-off value of the NLR. Several studies considered the prognostic implication of the NLR in terms of both NSCLC and SCLC [15,16,25,26]; however, the cut-off values vary across studies. Moreover, both neutrophils and lymphocytes are affected by several cancer-associated events, such as corticosteroid use as part of palliative treatment and coexisting infections. Further research is thus needed to identify the optimal NLR cut-off value for predicting the OS of patients with ED-SCLC.

We also examined the association between the number of prognostic factors and the incidence of irAEs. The incidence of irAEs was greater for patients without unfavorable prognostic factors, suggesting an association between the risk of irAEs and favorable prognosis. In NSCLC, the incidence of irAEs has been reported to be a favorable prognostic factor of ICI treatment [27,28,29]. In contrast, conflicting results have been reported regarding the association between irAEs and the outcome of ICI treatment for patients with ED-SCLC. Yokoo et al. assessed the development of irAEs and treatment efficacy for 40 patients with ED-SCLC treated with ICIs and platinum-plus-ETP regimens [30]. The median OSs were comparable between the irAE and non-irAE groups (27.6 vs. 24.9 months; *p* = 0.268). Nishimura et al. compared survival between patients with ED-SCLC who developed irAEs (*n* = 23) and those who did not (*n* = 67), and the median OS was longer for the patients with irAEs than those without irAEs (22 vs. 9.3 months, *p* = 0.013) [31]. Although it remains unclear whether the incidence of irAE is a prognostic factor in ED-SCLC, our results underscore the need for physicians to be aware of the risk of irAEs, especially in patients without liver metastasis and decreased albumin levels.

The limitations of the current study include the following. First, the current study was a retrospective cohort analysis with a limited sample size; hence, the validation of our findings using a prospective cohort is required. Second, the follow-up period in this study was relatively short. Third, the frequency of radiological evaluations varied among cases due to the retrospective nature of this study. Fourth, the proportion of patients with poor PS was relatively low, which may have influenced the results of this study. Finally, there is also a limitation regarding tumor markers. Neuron-specific enolase (NSE) is a useful tumor marker of SCLC, similar to ProGRP; however, NSE data were insufficient in this study because the NSE level was measured in a limited number of patients.

## 5. Conclusions

Liver metastasis and albumin levels at baseline were identified as independent unfavorable prognostic factors for patients with ED-SCLC undergoing chemoimmunotherapy regimens. Moreover, the number of prognostic factors was associated with both survival and the incidence of irAEs. Patients with both prognostic factors showed unfavorable OS, while patients without these factors were suggested to have a favorable prognosis but to be at a greater risk of irAEs.

## Figures and Tables

**Table 1:** Patient characteristics.

	Total (*n* = 130)	Poor (*n* = 35)	Moderate (*n* = 77)	Favorable (*n* = 18)	*p* Value
Age	71 (42–85)	73 (46–82)	69 (42–84)	70 (62–85)	0.67
Gender					
Male	103	29	61	13	
Female	27	6	16	5	0.67
Smoking status					
Never	4	1	3	0	
Former or current	126	34	74	18	0.69
Performance status					
0	27	1	22	4	
1	71	19	41	11	
2	23	9	12	2	
3–4	9	6	2	1	0.02
Clinical stage					
IIIB-IIIC	10	3	6	1	
IVA	36	6	22	8	
IVB	80	26	47	7	
Recurrent	4	0	2	2	0.10
Metastatic site					
Brain	29	12	13	4	0.12
Liver	40	17	20	3	0.03
Bone	25	5	15	5	0.73
Prior radiotherapy					
Yes	27	6	16	5	
No	103	29	61	13	0.67
Platinum doublet regimen					
Cisplatin and etoposide	6	1	5	0	
Carboplatin and etoposide	124	34	72	18	0.56
Immune checkpoint inhibitors					
Atezolizumab	98	25	57	16	
Durvalumab	32	10	20	2	0.34

**Table 2:** Results of univariate and multivariate analyses of overall survival.

	Univariate		Multivariate	
	HR (95% CI)	*p* Value	HR (95% CI)	*p* Value
Age (≥75 vs. <75)	1.43 (0.87–2.35)	0.16	1.66 (1.01–2.76)	0.06
PS (0–1 vs. 2–3)	0.89 (0.53–1.43)	0.59	0.79 (0.71–2.00)	0.50
Liver metastasis	1.81 (1.13–2.91)	0.01	2.03 (1.25–3.30)	0.002
NLR (<5.0 vs. ≥5.0)	0.60 (0.37–0.97)	0.04	0.97 (0.68–2.55)	0.07
Alb (<3.5 vs. ≥3.5)	2.02 (1.30–3.14)	<0.001	1.84 (1.14–2.96)	0.02

Abbreviations: CI, confidence interval; PS, performance status; NLR, neutrophil-to-lymphocyte ratio; Alb, albumin.

## Data Availability

The data used in the present study are available from the corresponding author upon request.
